# Artificial intelligence (AI) in medicine as a strategic valuable tool

**DOI:** 10.11604/pamj.2021.38.184.28197

**Published:** 2021-02-17

**Authors:** Andreas Larentzakis, Nik Lygeros

**Affiliations:** 1 First Department of Propaedeutic Surgery, Athens Medical School, National and Kapodistrian University of Athens, Hippocration General Athens Hospital, Athens, Greece,; 2 Laboratoire de Génie des Procédés Catalytiques, Centre National de la Recherche Scientifique/École Supérieure de Chimie Physique Électronique, Lyon, France

**Keywords:** Artificial intelligence, healthcare, medicine, neural network, deep learning, machine learning

## Abstract

Humans' creativity led to machines that outperform human capabilities in terms of workload, effectiveness, precision, endurance, strength, and repetitiveness. It has always been a vision and a way to transcend the existence and to give more sense to life, which is precious. The common denominator of all these creations was that they were meant to replace, enhance or go beyond the mechanical capabilities of the human body. The story takes another bifurcation when Alan Turing introduced the concept of a machine that could think, in 1950. Artificial intelligence, presented as a term in 1956, describes the use of computers to imitate intelligence and critical thinking comparable to humans. However, the revolution began in 1943, when artificial neural networks was an attempt to exploit the architecture of the human brain to perform tasks that conventional algorithms had little success with. Artificial intelligence is becoming a research focus and a tool of strategic value. The same observations apply in the field of healthcare, too. In this manuscript, we try to address key questions regarding artificial intelligence in medicine, such as what artificial intelligence is and how it works, what is its value in terms of application in medicine, and what are the prospects?

## Special feature

**Figure 1:**
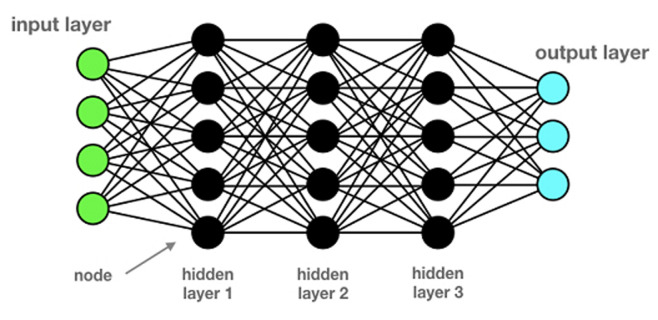
deep neural network architecture

It has always been an area of challenge for humans to create machines to outperform human capabilities in terms of workload, effectiveness, precision, endurance, strength, and repetitiveness. It is a way to transcend the existence and to give more sense to life, which is precious. The common denominator of all these creations was that they were meant to replace, enhance or go beyond the mechanical capabilities of the human body. This path of evolution is smooth and predictable. This story takes a different shift or another bifurcation to be more precise when Alan Turing introduced the concept of a machine that could achieve human-level performance in thinking in 1950 [1]. However, the revolution began with the computational model for neural networks (NNs) with Warren McCulloch and Walter Pitts, and this time the evolution is unpredictable [2]. In mathematical terms, the network forms a directed, weight graph. This point of view was reinforced by Norbert Wiener, who introduced the feedback [3]. Artificial neural network (ANN) started at first level as an attempt to exploit the architecture of the human brain to perform tasks that conventional algorithms had little success with. Artificial neural network architecture is based on nodes arranged in layers and connected via their input(s) and output(s), in a way attempting to imitate brain neurons activity (Figure 1). Artificial intelligence (AI) as a term describes the use of computers to imitate intelligence and critical thinking comparable to humans, and it was first mentioned by John McCarthy during a conference held in 1956 [4].

**Figure 2:**
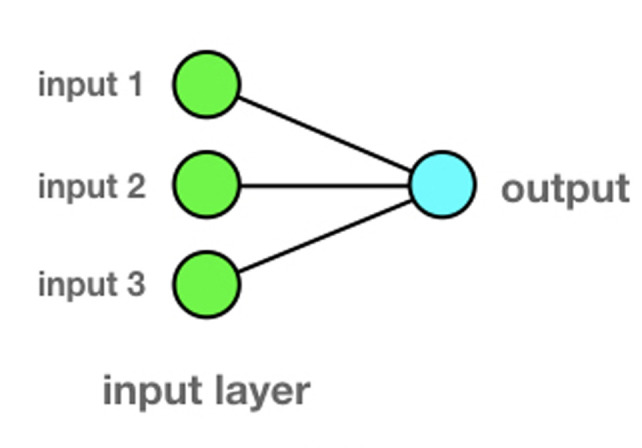
single artificial neuron with three inputs and one output

**How it works:** but how it works? Let us take a brain neuron; if the incoming synaptic stimuli (inputs) are of sufficient intensity, then the neuron will fire (output). Figure 2 shows a model of a single artificial neuron with three inputs and one output. Inputs and outputs are “0” or “1”. In order to keep things simple, the following example will not use a firing threshold. We want to train the neuron according to the follo}}wing pattern:

Case: [input a - input b - input c] ➝[output]

Case A: [0 - 0 - 0] ➝[0]

Case B: [0 - 0 - 1] ➝[1]

Case C: [1 - 1 - 0] ➝[0]

Case D: [1 - 0 - 0] ➝[0]

The first step is to weight each case input by multiplying it with a random positive or negative number. Then we add all the weighted inputs of each case. Next thing, we normalize this sum of each case by using a sigmoid function in order to get a result between 0 and 1, as an output of the neuron for each case. Now we calculate the error between the normalized sum and the actual training output of each case; we use this error to adjust the weights to be used for the next round of calculations. Adjusting the weights takes under consideration the input, the calculated output, and the magnitude of the error, in a way that the adjustment to be proportional to the magnitude of the error (sigmoid curve gradient works for this). By repeating this cycle thousands of times, the neuron finally makes almost no adjustments to the weights of the inputs, meaning that it has been trained to recognize the pattern. Now one may introduce a new set of three inputs, and the already trained algorithm will be able to provide an output that corresponds to the already recognized pattern. Thus, one could summarize that artificial thinking is a pattern recognition by weighting, comparing, and adjusting many many times before a pattern replication output is created.

A sophisticated AI algorithm needs to be exposed to data feeds, which are structured and labelled in a way the algorithm can recognize (i.e. numbers, pixels, colours). Ng and Dean, Stanford and Google, respectively, leaders on computer science, created an ANN that learned to recognize higher-level concepts, such as human face, human body, or animals [5]. Unsupervised pre-training, increased computing power from multiple graphics process units (GPUs), and distributed computing allowed the use of larger (increased number of nodes) and deeper (increased number of layers) networks, particularly in image and visual recognition tasks, which became known as deep learning (DL) [6-8]. And now at high-level research, we use deep neural networks (DNNs) with tensor processing units (TPUs) [9]. Nowadays, the most representative area of thinking machines evolution has been the world of strategy board games. Board games, such as chess, shogi or go, are considered an expression of human intellect at the highest level; however, DNNs as AlphaGo, AlphaGo Master, AlphaGo Zero mastered all those sharp games [10-13]. The 3D models of proteins that AlphaFold generates are far more accurate than any that have come before marking significant progress on one of the core challenges in biology [14, 15].

**Artificial intelligence applications in healthcare:** machine learning (ML) algorithms based on NNs have already been used in the field of healthcare, mainly in medical diagnosis and prognosis, disease treatment, drug development, gene editing, and personalized medicine.

**Disease diagnosis and prognosis:** medical imaging plays a key role as an input. Plain film X-rays have been widely used as inputs in ML algorithms to teach them to diagnose lung conditions, such as pneumonia, emphysema, and tuberculosis or to detect bone age, maturity, and fractures [16-20]. Neural networks, fed with chest computed tomography (CT) scans from smokers can identify and stage chronic obstructive pulmonary disease as well as predict mortality [21]. In the field of ophthalmology, AI-based algorithms have been utilized for fundus screening in diabetic patients, age-related macular degeneration, and congenital cataract diagnosis [22-27]. Cancer diagnosis is another field that ML and NNs have been tested and proved to be superior or non-inferior to humans, including malignancy detection in pathology images, in screening mammography, in CT or magnetic resonance imaging (MRI) or positron emission tomography (PET) scans, and skin clinical images [28-40]. Also, ML algorithms fed with endoscopic images and videos reached human-like performance in gastrointestinal neoplasms detection, such as of oesophagal cancer, gastric cancer, and large bowel polyps [41-43]. Furthermore, cardiologists are investigating the ML NNs algorithms in the diagnosis, severity classification, and prognosis of cardiovascular diseases, by processing data obtained from electronic health records (EHR), electrocardiography, echocardiography, coronary artery calcium scoring, coronary CT angiography, and MRI [44-47]. For example, AI models can predict survival outcomes given a specific diagnosis, such as pulmonary hypertension by 3D cardiac MRI processing [48]. Many studies are also existing in the field of neuroscience. Deep neural networks (DNNs) can predict the future diagnosis of autism in high-risk children by processing brain magnetic resonance imaging (MRI), assess the progression of dementia by processing a single amyloid PET scan, detect intracranial haemorrhage on CTs, as well as to diagnose schizophrenia and predict the risk of suicide by the processing of functional MRIs (fMRIs) and EHR [49-54]. Finally, timely diagnosis of infectious diseases in terms of pathogen identification and antibiotic susceptibility testing is feasible through ML processing of bacterial Raman spectra or bacterial and viral mRNA [55,56].

**Disease treatment:** in the field of psychiatry, researchers used functional magnetic resonance imaging or functional (fMRI) and proton magnetic resonance spectroscopy (^1^H-MRS) as inputs to a linguistic AI platform; as a result, they were able to manage lithium dosage in bipolar patients [57]. In another study, AI virtual interviewer could capture more post-traumatic stress symptoms from veterans than the human interviewers [58]. Moreover, in the field of surgery, as surgical robots are already here, artificial intelligence short guide ribonucleic acid implementation in operations is already happening in experimental and dental settings [59,60].

**Drug development:** the development of a new drug is a costly and time-consuming process, which includes identification of targets for intervention, hypothesis for a new compound, and clinical trials of level I, II, and III [61]. The recognition of a possible target and the hypotheses generation for a new compound relies on pattern recognition. Chemists are skilled to recognize such patterns, relate them to retrosynthetic analysis, and predict the properties, absorption, distribution, metabolism, excretion, and toxicity (ADMET). Deep learning architecture algorithms are up-and-coming tools in the field of drug development because they imitate chemists' pattern recognition skills. Moreover, it seems possible to advance the whole process to a next level by being able to de novo design of drugs, considering all the available domain, ligand-based, and associations data during the development of a model [62,63]. The most successful paradigm of such an effort is the discovery of a new type of antibiotic, halicin, that has a different structure from known antibiotics and a broad-spectrum antibacterial activity including resistant strains such as pan-resistant *Acinetobacter baumannii*. The same DL algorithm was able to identify eight compounds with antibacterial activity and different structure comparing with the already known antibiotics [64]. Even if the main focus of DL-aided drug innovation is on small molecules, some approaches utilize DL to design proteins and develop antibodies [14,15,65].

**Biomarkers:** the principles, approaches, and tools used in drug development are applied to the identification of biomarkers, which are molecules that when found in body fluids or tissues are pathognomonic, i.e. they provide absolute certainty for disease diagnosis. Biomarkers are useful in imaging, early diagnosis, prognosis, disease progression evaluation, risk assessment for developing a specific disease, and predicting patients' response to a drug. Pembrolizumab for malignancies carrying a specific genetic biomarker is an example of how AI-aided biomarker identification could lead to the development of targeted biotherapies [66]. There are more other AI biomarker studies like Tasaki *et al*. regarding drug responses for patients with rheumatoid arthritis, or like Khera *et al*. on genome-wide polygenic scores as a risk assessment to develop coronary artery disease, type 2 diabetes, atrial fibrillation, inflammatory bowel disease, or breast cancer [67,68].

**Gene editing:** gene editing biotechnology of clustered regularly interspaced short palindromic repeats (CRISPR) and its associated protein 9 (Cas9) uses short ribonucleic acids (RNAs) as guides (sgRNA) to target a specific deoxyribonucleic acid (DNA) location in order to cut and edit it. These guides, however, may fit DNA locations other than the desired target resulting in the so-called off-target effect. Thus, the selection of the sgRNA molecules to be used is of significant importance. Machine learning algorithms have proved to be promising in the identification of such molecules caring the lowest possible off-target propensity for specific DNA targets [69,70].

**Personalized medicine:** patients´ symptoms, signs, and test results have to be evaluated by a physician or a multidisciplinary team of experts before a treatment plan is suggested. International Business Machines Corporation (IBM) AI platform “Watson” was initially made known by winning a television quiz show competition. In a study by Wrzeszczynski *et al*., Watson managed, in 10 minutes, to deliver a treatment plan for a glioblastoma case comparable to the plan that experts made in 160 hours [71]. In another study, Watson was able to suggest cancer therapeutic options that oncologists had overlooked [72]. It seems that if AI systems are provided with large enough amount of data, then they may outperform human physicians in diagnoses or treatment plans. The challenge becomes more intense when big data, such as omics, microbiome sequencing, EHR, social media, and digital images and videos are implemented to the patients' care. Big data are heterogeneous and continuously adding up. As a result, it is difficult for humans to manually analyze them in an effective and meaningful manner in t he field of healthcare. In contrast, AI has the potential to undertake and deliver this task. Interesting approaches are the web-based AI platforms or AI smartphone applications which answer patients´ questions, provide them with advice on whether their condition requires medical attention, and monitor adherence to medications [73,74].

## Conclusion

Artificial intelligence research is expanding, and there are increasing AI applications in medicine, too. It is a quickly evolving new era given that DL algorithms seem to perform better than statistics or humans, especially when it comes to big data. Artificial intelligence is a valuable tool, firstly and most importantly, for people and their healthcare. As such, physicians and healthcare systems will embrace, adapt, and evolve accordingly. It is becoming more and more apparent that AI will eventually create the pre- and post- AI era in medicine, too.
